# Improved methods for total and chloroplast protein extraction from *Cajanus* species for two-dimensional gel electrophoresis and mass spectrometry

**DOI:** 10.1371/journal.pone.0308909

**Published:** 2024-08-15

**Authors:** Arunima S., Alakesh Das, Prakash Jyoti Kalita, Rahul Ishwar Patil, Neha Pandey, Mamta Bhattacharjee, Bidyut Kumar Sharma, Debajit Das, Sumita Acharjee

**Affiliations:** 1 Department of Agricultural Biotechnology, Assam Agricultural University, Jorhat, Assam, India; 2 DBT-North-East Centre for Agricultural Biotechnology, Assam Agricultural University, Jorhat, Assam, India; Mahatma Phule Krishi Vidyapeeth College of Agriculture, INDIA

## Abstract

The recent advances in pigeon pea genomics, including high-quality whole genome and chloroplast genome sequence information helped develop improved varieties. However, a comprehensive *Cajanus* proteome, including the organelle proteome, is yet to be fully mapped. The spatial delineation of pigeon pea proteins at sub-cellular levels and inter-organelle communication could offer valuable insights into its defense mechanism against various stresses. However, the major bottleneck in the proteomic study is the lack of a suitable method of protein extraction and sample preparation compatible with two-dimensional gel electrophoresis (2D-PAGE), liquid chromatography-mass spectrometry (LCMS), or matrix-assisted laser desorption ionization-time of flight (MALDi-ToF). Our study introduces two efficient methods, one for isolating total proteins and another for organelle (chloroplast) proteins from various *Cajanus spp*. For total protein extraction, we have optimized a protocol using phenol in combination with a reducing agent (DTT) and protease inhibitor cocktail, also washing (6–7 times) with ice-cold acetone after overnight protein precipitation of total proteins. Our modified extraction method using phenol for total leaf protein yielded approximately 2-fold more proteins than the previously reported protocols from *C*. *cajan* (3.18 ± 0.11 mg/gm) and *C*. *scarabaeoides* (2.06 ± 0.08 mg/gm). We have also optimized a protocol for plastid protein extraction, which yielded 1.33 ± 0.25 mg/10 gm plastid proteins from *C*. *cajan* and 0.88 ± 0.19 mg/10 gm plastid proteins from *C*. *scarabaeoides*. The 2D-PAGE analysis revealed 678 ± 08 reproducible total protein spots from *C*. *cajan* and 597 ± 22 protein spots from *C*. *scarabaeoides*. Similarly, we found 566 ± 10 and 486 ± 14 reproducible chloroplast protein spots in *C*. *cajan and C*. *scarabaeoides*, respectively. We confirmed the plastid protein fractions through immunoblot analysis using antibodies against LHCb1/LHCⅡ type Ⅰ protein. We found both methods suitable for 2D-PAGE and mass spectrometry (MS). This is the first report on developing protocols for total and chloroplastic protein extraction of *Cajanus* spp. suitable for advanced proteomics research.

## Introduction

Pigeon pea is a globally important legume which widely consumed in India as a vegetable protein and is considered as a source of essential nutrients such as vitamins, minerals, and vital amino acids [1]. India emerges as a major contributor of pigeon pea production with 4.90 million hectares area under cultivation accounting for 4.22 million metric tons of production [2]. The crop faces stagnant productivity due to biotic and abiotic stresses. To overcome this, there exists a concerted effort to enhance genomic resources and employ innovative methodologies aimed at augmenting yield, improving nutritional quality, and securing resilience against both abiotic and biotic stresses [3, 4]. A high-quality pigeon pea genome analysis using the Next-generation sequencing platforms has led to the annotation of 48,680 protein-coding gene models from an 833.07 Mbp genome sequence with 1,213 disease resistance/defense responsive gene [5, 6]. The genome of cultivated pigeon peas provides insight into legume karyotypes, polyploid evolution, and crop domestication. Organelles like chloroplast play an important role in plant defense. Chloroplasts (cp), specialized organelles inherent to photosynthetic eukaryotes, play a pivotal role in growth and developmental processes. In the recent past, a draft chloroplast genome of *C*. *cajan* and *C*. *scarabaeoides* was generated [7]. The cp genome of *C*. *cajan* is 152,242 bp long and *C*. *scarabaeoides* is 152,201bp long with 116 unique genes, including 30 tRNA, 4 rRNA, 78 predicted protein coding genes, and 5 pseudogenes. However, an equivalent full map of the pigeon pea proteome, especially the plastid proteome, remains elusive. The information on pigeon pea proteome can be used as a valuable reference for the identification of biomarkers from various tissues of pigeon pea. Delineating the spatial distribution of all pigeon pea proteins at the organ, tissue, and cellular levels can offer insight into the pathogen/pest resistance proteins and other stresses.

The advent of advancements in techniques like mass spectroscopy (MS), label-free quantification, and isobaric tags for relative and absolute quantitation (iTRAQ) and several bioinformatics approaches have revolutionized the field of proteomics and encouraged researchers to extensively study the global protein accumulation in several plants [8]. Although, these new improvements have advantages like increased sensitivity, the old gel-based techniques like 2D-PAGE and differential in-gel electrophoresis (DIGE) are still valuable for visualizing protein gels, detecting protein spots in abundance and extracting information about protein isoforms [9]. Amongst the different techniques used to study proteomics, 2D-PAGE is one of the important proteomic tools used for the analysis and comparison of synthesis, turnover, and modification of proteins under various environmental stresses [10].

The possibility of creating a uniform technique for protein extraction from various tissues of taxonomically distinct plant species has been met with minimal success. As a result, there have been worldwide initiatives to create simpler protocols for optimum protein extraction from various tissues of different plant species [11, 12]. Methods like TCA-acetone-based protein extraction are generally regarded as being efficient for younger tissues [13, 14] while highly recalcitrant tissues have been reported to respond better to phenol extraction followed by Ammonium acetate precipitation in methanol [15]. The TCA-acetone extraction method and phenol extraction method have already been successfully reported in pigeon pea. However, the protein yield was low (1–2 mg/g leaf sample) from pigeon pea (*Cajanus cajan*, variety ASHA, ICPL-87119). Also, protein spots identified were less (250–400) through these methods [16]. However, the available protocols remain restricted to certain genotypes and the knowledge regarding the protein extraction in wild genotypes of pigeon pea is rather sparse. It has been further reported that the total protein content of pigeon pea wild varieties is lower and the phenolic and tannin contents are higher than cultivated varieties [17], which are known to interfere with the protein processing. Thus, the optimization of an extraction buffer and protocol is required for obtaining good quality protein from different genotypes of pigeon pea that is compatible with 2D-PAGE, LCMS, and MALDi-ToF.

Similarly, it is challenging to analyse the complete chloroplast proteome via global strategies due to the significant amount of membrane proteins that are hard to solubilize [18]. Most of the chloroplast protein extraction protocol requires highly sophisticated instruments like ultra-centrifuge and controlled conditions. There are only limited established approaches to separate complete proteins from chloroplasts. Although, chloroplast protein extraction has been reported in plants like *Arabidopsis* [19], spinach [20, 21] and tomato [22] using the percoll-based density gradient centrifugation, however, no protocol is available for pigeon pea. Our study aims to present an optimized protocol for extraction of total protein and chloroplast protein from wild as well as cultivated species of pigeon pea which is compatible with downstream proteomics techniques like SDS-PAGE, 2D-PAGE, and MS analysis.

## Materials and methods

### Plant material

The Pigeon pea cultivar ICPL-332 (*C*. *cajan*) and the wild-type genotype ICP-15738 (*C*. *scarabaeoides*) were obtained from the International Crops Research Institute for Semi-Arid Tropics (ICRISAT), Patencharu, Hyderabad, India. Seeds upon sterilization with 70% ethanol were germinated under dark over filter paper inside petri plates containing adequate moisture at 25±3°C for 3 days. Germinated seedlings were transferred to pots containing a potting mixture of sand: soil: and vermicompost (1:1:1) and grown inside a phytotron unit, AAU Jorhat at 28±2°C, 60–70% relative humidity and under a constant long day photoperiod of 16 h light/8 h dark. Plants were watered at regular intervals and fertilizers were applied every two weeks. For total and chloroplast protein extraction fresh leaves were collected from adult plants of 5 and 10 weeks old, respectively (**S1 Fig**).

### Total protein extraction

To extract proteins from pigeon pea leaf tissues following methods were used.

#### Tris-chloride extraction method

In this method, the total proteins from pigeon pea were extracted using the Tris-chloride extraction buffer followed by trichloroacetic acid (TCA) precipitation [16]. The leaf tissues were finely crushed with a micro pestle and were resuspended in Tris extraction buffer (1M Tris-Cl, pH 8.0, 1M NaCl, 2mM EDTA, 25 mM DTT, and protease inhibitor cocktail (PIC)) in a ratio of 1:5 v/v. The cell extract was then centrifuged twice at 10,000 rpm and 4°C for 10 minutes to remove the cell debris. The supernatant was transferred to a fresh tube and the protein was precipitated by adding precipitation buffer containing acetone and TCA, in a ratio of 1:8:1 v/v followed by overnight incubation at -20°C. After overnight incubation, the tubes were centrifuged at 11,500 rpm and 4°C for 15 minutes. The supernatant was discarded and the pellet was washed with 300 μl of ice-cold acetone, this step was repeated 6–7 times to ensure the complete removal of residual salt present in the solution. Each washing step was followed by centrifugation at 11,500 rpm and 4°C for 5 min. After completion of the washing steps, the pellet was dried at room temperature for 5–10 minutes. The dried pellet was then solubilized in 20 μl ReadyPrep™ 2-D starter kit Rehydration/sample buffer (Bio-Rad, USA) and quantified using the Bradford method for further use [23].

#### Phenol extraction method

The phenol extraction method was used in the case of pigeon pea wild genotype for total protein extraction with minor modifications. A total of 1 g of fresh leaf tissues were finely grounded with liquid nitrogen and resuspended in extraction buffer (0.7 M Sucrose, 0.5 M Tris base pH 8.0, 30 mM HCl, 50 mM EDTA, 0.1 M KCl, 25 mM DTT and PIC) in a ratio of 1:3 v/v. The cell extract was vortexed vigorously and an equal volume of Tris buffered phenol was added followed by vortexing and incubation at 4°C for 10 min. The phenolic phase was recovered after centrifugation at 13000 rpm and 4°C for 15 minutes. The phenolic phase was mixed with an equal volume of extraction buffer, vortexed, and centrifuged at 13000 rpm and 4°C for 15 minutes. For protein precipitation, 0.1 M Ammonium acetate in cold methanol was added in a ratio of 1:5 v/v to the phenolic phase and, after proper mixing, was incubated overnight at -20°C. After overnight incubation, the protein pellet was recovered by centrifugation at 13000 rpm and 4°C for 15 minutes followed by washing thrice with 0.1 M Ammonium acetate solution and twice with acetone. The pellet was air-dried and resuspended in 200 μl of ReadyPrep™ 2-D starter kit Rehydration/sample buffer (Bio-Rad, USA) and quantified using the Bradford method.

### Chloroplast extraction

**Fig 1:**
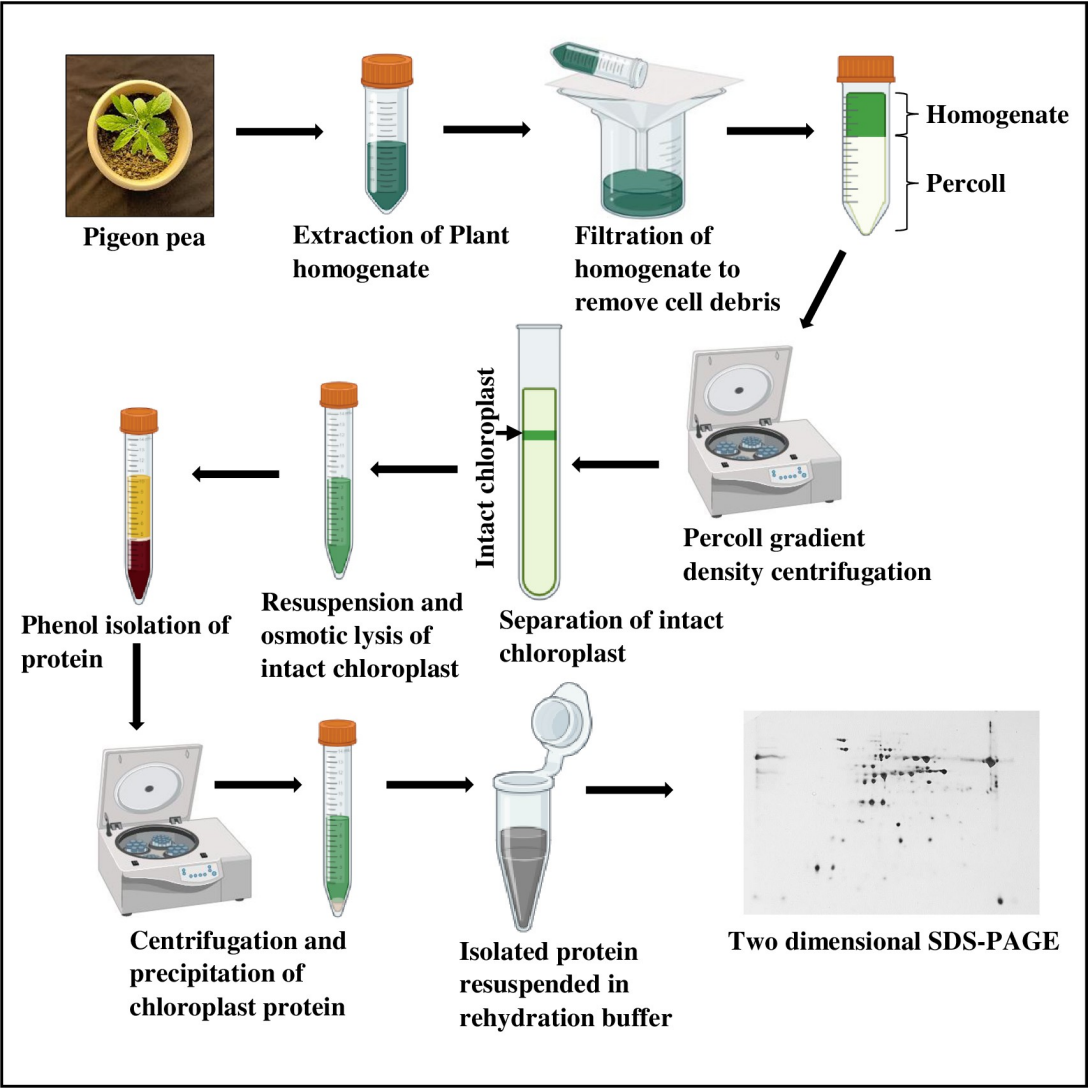
Graphical representation of entire workflow which was followed for the isolation of chloroplast proteins from pigeon pea genotypes *C*. *scarabaeoides* and *C*. *cajan*.

Pigeon pea chloroplasts were isolated using a method [19] with modifications (**Fig 1**). For isolation of chloroplast, around 10 g of freshly collected leaf samples were processed in grinding buffer [50 mM HEPES-KOH (pH 8.0), 330 mM sorbitol, 2 mM EDTA-Na_2_ (pH 8.0), 5 mM ascorbic acid, 5 mM cysteine and 0.05% bovine serum albumin (BSA)] with 2–4 blender strokes within 5 sec to obtain a coarse macerate. The macerate was then filtered through a double layer of muslin cloth. The filtered macerate was centrifuged at 3400 rpm and 4°C for 3 minutes to pellet down the chloroplast fraction. The pellet was resuspended in 2–3 ml of wash buffer [50 mM HEPES-KOH (pH 8.0), 330 mM sorbitol, and 2 mM EDTA-Na_2_ (pH 8.0)] and loaded on the percoll gradient 40%/85% followed by centrifugation at 3750×g and 4°C for 10 min. Intact chloroplasts were collected from the interphase of 40%/85% percoll which was subsequently diluted in 2 ml of wash buffer. The resuspended chloroplast pellet was centrifuged at 1200×g for 3 min and the supernatant was discarded to obtain the purified intact chloroplast.

### Estimation of chlorophyll concentration

Chlorophyll concentration was estimated by using the protocol described by Arnon *et al*., [24]. For total chlorophyll determination, 10 μl of purified chloroplast was mixed with 1 ml of 80% acetone. The mixture was centrifuged at 3000×g for 2 min. and the supernatant was used to measure the absorbance at 652 nm (A_652_). The yield of the chlorophyll was calculated using the formula:

$$(mg\;chlorophyll/ml)=(A_{652}\times 100)/36$$ (1)

### Estimation of intact chloroplasts

The ferricyanide photoreduction assay was conducted based on a protocol described by Lilley *et al*., [25] with modification. Two chloroplast solutions were prepared, one without osmotic shock and the second with osmotic shock. For the chloroplast solution without osmotic shock (A), a volume of chloroplast equivalent to 100 μg chlorophyll was mixed with 4 ml of wash buffer and 60 μl of 100 mM potassium ferricyanide. Similarly, for the preparation of chloroplast solution with osmotic shock (B), chloroplast equivalent to 100 μg chlorophyll was mixed first with 2 ml of distilled water followed by an incubation period of 15 sec to allow osmotic shock immediately after which 2 ml of wash buffer along with 60 μl of 100 mM potassium ferricyanide was added. The samples were then kept inside a beaker containing ice-cold water and illuminated with a closely held 40 W bulb. Absorbance was measured before illumination and then after every 2 min absorbance was measured till 6 min and plotted on the graph against time on the X axis. The rate of decrease in absorbance of each sample was used to calculate the slope of the graph and the percentage of intact chloroplast was calculated using the formula:

[(B‐A)/B]×100%=%intactchloroplasts (2)

### Chloroplast protein extraction and quantification

Protein extraction from chloroplast was done by modified Wang and his co-worker’s [26] protocol. For protein extraction, the chloroplast pellets were resuspended in 1.5 ml of resuspension buffer (100 mM Tris, 100 mM EDTA, 50 mM borax, 50 mM ascorbic acid, 1% Triton X-100, 2% β-mercaptoethanol and 30% sucrose) and it followed by vigorous vortexing for 10 min at room temperature. About 1.5 ml of tris-saturated phenol was added to the mixture and vortexed again for 10 min. The tubes were centrifuged at 11573 rpm and 4°C for 15 minutes and the upper phenol phase was transferred to new tubes. Protein precipitation was done with Ammonium sulfate-saturated methanol followed by overnight incubation at -20°C. After overnight incubation, the tubes were centrifuged at 11573 rpm and 4°C for 15 minutes. The supernatant was discarded, and the pellet was first rinsed with ice-cold methanol followed by 3–5 times washing with acetone. Finally, after discarding acetone the pellets were air dried for 5–10 minutes, and then resuspended in 100μl of ReadyPrep™ 2-D starter kit Rehydration/sample buffer (Bio-Rad, USA). Protein quantification was done with the Bradford method and the quantified protein was used for 2D-PAGE.

### Catalase enzyme assay

The catalase enzyme assay was performed as described by Pandey *et al*., [27]. Around 10 μg of both total and chloroplast proteins were mixed with 10 μl of H_2_O_2_ and the absorbance was measured at 240 nm (A_240_) at 1 min interval for five minutes. The absorbance of the solution without H_2_O_2_ served as the reference blank, and a graph was plotted against time.

### Immunoblot analysis

Immunoblot analysis was performed using a protocol described by Zienkiewicz *et al*., [28]. Around 20 μg of total and chloroplast proteins were used to perform one-dimensional electrophoresis (SDS-PAGE). The protein was transferred from gel to nitrocellulose membrane and the membrane was blocked using 5% BSA for 1 h at room temperature. Then the membrane was incubated overnight with rabbit-raised polyclonal LHCb1/LHCⅡ type Ⅰ chlorophyll a/b binding protein primary antibody at a dilution of 1:2500 (Agrisera, Sweden). After washing and removal of the unbound primary antibody, the membrane was incubated for 1 h at a dilution of 1:10,000 with the goat-raised polyclonal secondary antibody conjugated with alkaline phosphatase (AP) enzyme, which can bind with the heavy chains of rabbit IgG (Promega, US). BCIP tablets were used as substrate for alkaline phosphatase conjugated with the secondary antibody and the reaction was conducted for 15 min. followed by image capture and analysis.

### Two dimensional SDS-PAGE

The total and chloroplast proteins from pigeon pea leaves were first resolved based on isoelectric points. For passive rehydration (50 and 250 μg) proteins mixed with 2 μl bromophenol blue and rehydration buffer to a final volume of 125 μl were loaded onto the immobilized pH gradient (IPG) strips (3–10 & 4–7) of 7 cm length for 16 h at 20°C (ReadyStrip™ IPG Strips, Bio-Rad, USA) inside PROTEAN®i12™ IEF system (Bio-Rad, USA). After completion of passive rehydration, isoelectric focusing (IEF) was performed according to the manufacturer’s guidelines by following the program mentioned (**Table 1**).

**Table 1:** PROTEAN®i12™ IEF system condition for IEF.

Step	Voltage	Gradient	Current	Value	Units
1	250	Linear	50	0:20	HH:MM
2	4000	Linear	50	2:00	HH:MM
3	4000	Rapid	50	10,000	Volt Hr

After IEF, the strips were equilibrated with 1.5 ml equilibration buffer I (6M Urea, 2%SDS, 0.375M Tris-Cl, 20% glycerol and 130mM DTT) and equilibration buffer II (6M Urea, 2%SDS, 0.375M Tris-Cl, 20% glycerol and 135mM Iodoacetamide) for 15 min each. After equilibration, the strips were rinsed in 1X tank buffer and loaded over a 12% resolving gel followed by sealing with low melting agarose containing bromophenol blue. Upon completion of gel electrophoresis, the protein spots were visualized by silver staining and the image was captured over a trans-illuminator under Epi-white light and was used for further analysis [29, 30].

#### Mass spectrometric analysis

Ten random protein spots were selected from different regions of the gel and the selected protein spots were manually excised from the gel. The MS/MS analysis for identification of protein spots was outsourced to a commercial service provider, Valerian Chem Private Limited (New Delhi, India). The spots were destained using the destaining solution and reduced with 5 mM tris (2-carboxyethyl) phosphine (TCEP). The protein was further alkylated with 50 mM iodoacetamide and then digested with trypsin (1:50, trypsin/lysate ratio) for 16 h at 37°C.

Mass spectrometric analysis of peptide mixtures was performed on an Easy-nlc-1000 system coupled with an Orbitrap Exploris mass spectrometer. About 1 μg of peptide sample was loaded on C18 column, 3.0 μm Acclaim PepMap (Thermo Fisher Scientific, USA) and the peptide separation was achieved with solvent A (2% acetonitrile, 0.1% formic acid) and solvent B (80% acetonitrile, 0.1% formic acid) at a flow rate of 0.8mL/min. All samples were processed, and raw files generated were analyzed with Proteome Discoverer (v2.5) against the UniProt *Cajanus cajan* database. For dual Sequest and Amanda search, the precursor and fragment mass tolerance were set at 10 ppm and 0.2 Da, respectively. Both peptide spectrum match (PSM) and protein false rate (FDR) were set to 0.01 FDR.

## Results and discussion

Extraction of good quantity and quality proteins is the key to proceeding with proteomic research. The protein extraction from crops such as pigeon pea having high phenols and tannins content impedes the process. Therefore, optimizing a protein extraction protocol is essential to conduct proteomic research.

### Protein enrichment and quality enhancement of pigeon pea total leaf protein

Total protein extraction methods from pigeon pea were previously reported [16], however, we observed a very low yield of total proteins that is 0.42 ± 0.09 and 0.98 ± 0.53 mg/g from TCA-Acetone precipitation and Phenol extraction method respectively in case of the wild genotype (**Table 2**). The low yield can be attributed to the presence of high content of tannins and phenolic compounds which affects the downstream extraction and quantification [17]. The extraction buffer suggested in the method given by Singh *et al*., [16] used β-Mercaptoethanol which is comparatively a weak reducing agent [31] and is not efficient enough to remove the tannins and phenolic compounds from the wild genotype. Hence, to overcome this problem we used Dithiothreitol (DTT) as a reducing agent which is a much stronger reducing agent [32] in the extraction buffer. In our modified extraction buffer, we used an optimized concentration of DTT (25 Mm). Further, in the extraction buffer, Singh *et al*., [16] used PMSF which acts against only serine proteases [33] apart from this it has a very short half-life of just 35 min when 20 mM was used in an aqueous solution at pH 8.0. In the modified method, we used a protease inhibitor cocktail (PIC) (Cat. P9599, Sigma) which has a broad specificity of inhibitory properties against serine, cysteine, aspartic metalloproteases, and aminopeptidases. Also, it remains active for a relatively longer period hence, can effectively control protein degradation [34]. We observed that the protein extracted with the extraction buffer suggested by Singh *et al*., [16] led to improper protein separation during IEF which ultimately resulted in multiple horizontal/vertical streaks (**S2 Fig**). The improper IEF run resulted from the accumulation of high amounts of tannins, phenolic compounds [35], and Ammonium salts (used in protein precipitation) among which the modified extraction buffer was able to remove tannins and phenolic compounds but for removal of Ammonium salts we increased number of washings. With all these changes we were able to increase the overall recovery and improve the quality of protein which is suitable to perform any proteomics research.

**Table 2:** Comparison of total protein yield before or after modification in the extraction buffer.

Pigeon pea genotypes	Total protein yield (mg/g) without modifications		Total protein yield (mg/g) with modifications	
	TCA-acetone Method	Phenol extraction Method	Tris-Cl Method and TCA-acetone precipitation	Phenol extraction Method
ICPL-332	1.63 ± 0.22	3.07 ± 0.54	1.89 ± 0.34	3.18 ± 0.11
ICP-15738	0.42 ± 0.09	0.98 ± 0.53	0.86 ± 0.04	2.06 ± 0.08

However, in the cultivated genotype although the protein yield was comparable with the original method, the quality and purity of the extracted proteins were improved as no vertical/horizontal streak appeared on the 2D-PAGE gels. Altogether, our data suggest that the method given by Singh *et al*., 2015 is suitable for only *C*. *cajan* (ICPL-87) however, our protocol was found to be suitable for not only enriching the total yield but also for extracting low conductivity high-quality total leaf proteins from *Cajanus* leaves, irrespective of the species.

### Quantity of protein for separation

**Fig 2:**
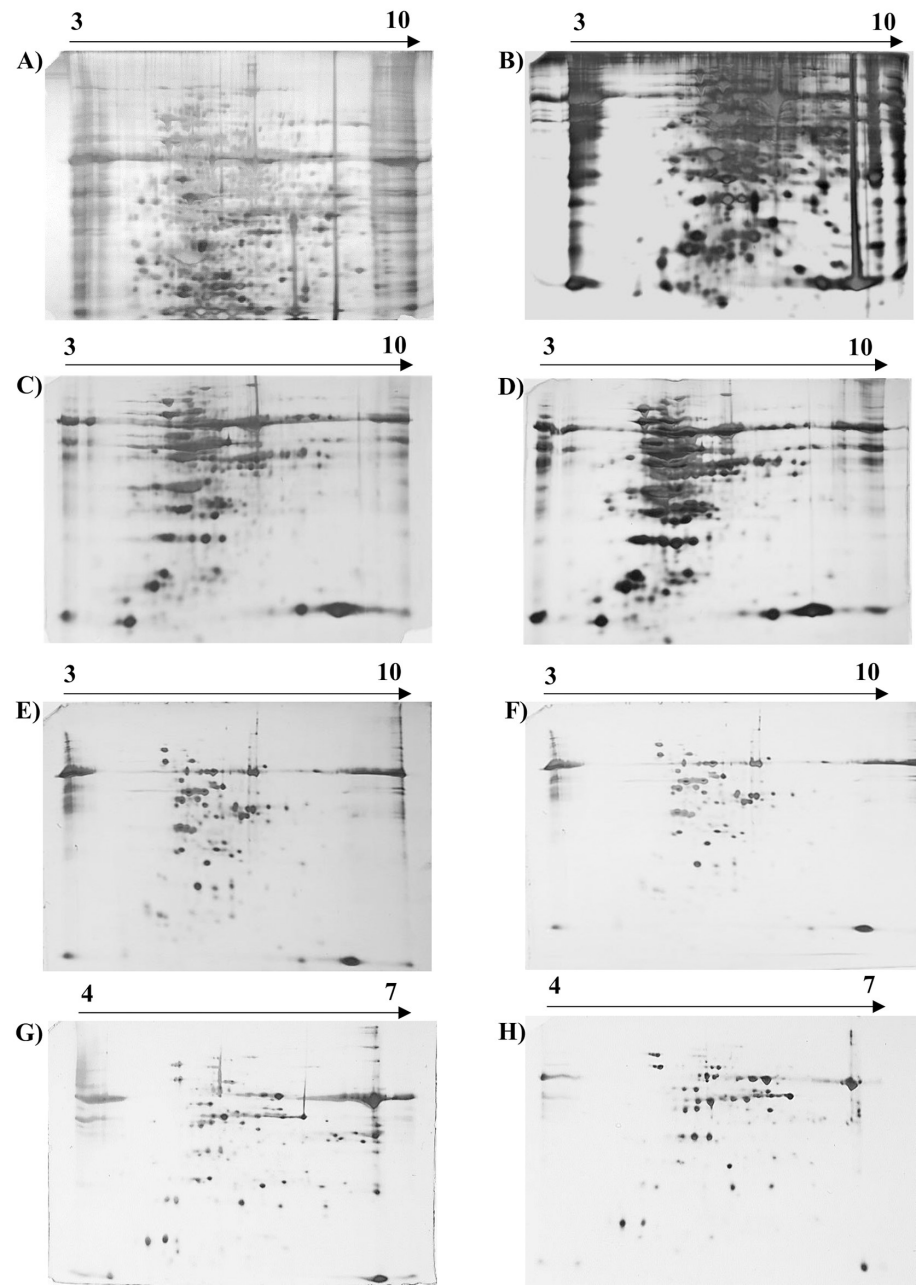
Two dimensional SDS-PAGE analysis of total proteins from pigeon pea genotypes *C*. *scarabaeoides* and *C*. *cajan*. A-B) Two hundred and fifty micrograms of total proteins from pigeon pea genotypes *C*. *cajan* and *C*. *scarabaeoides* were electrophoresed on 12% SDS-PAGE gel. C-D) Hundred microgram of total proteins from pigeon pea genotypes *C*. *cajan* and *C*. *scarabaeoides* and were electrophoresed on 12% SDS-PAGE gel. E-F) Fifty microgram of total proteins from pigeon pea genotypes *C*. *cajan* and *C*. *scarabaeoides* and were electrophoresed on 12% SDS-PAGE gel. G-H) Fifty microgram of total proteins from pigeon pea genotypes *C*. *cajan* and *C*. *scarabaeoides* were electrophoresed on 12% SDS-PAGE gel (IPG strip pH gradient range is 3–10 & 4–7 for A-F & G-H, respectively).

We loaded a range of protein quantities starting from 50 μg to 250 μg in the IPG strips to identify the optimum amount of protein required to obtain good quality 2D-PAGE gels. Initially, 250 μg of total proteins were subjected to 2D-PAGE analysis by resolving the proteins over the 7cm IPG strips of a 3–10 pH gradient range (Bio-Rad, USA). Even though we were able to obtain around 500–700 protein spots, it remained difficult to analyse the proteins and to excise individual protein spots from the gel for further analysis due to the large number of protein spots. We reduced the concentration to 50 μg and resolved the proteins over IPG strips of 3–10 pH and 4–7 pH in the case of total proteins followed by 2D-PAGE analysis as reported by previous workers [36–38]. Reducing the concentration resulted in a significant increase in the number of distinct protein spots, which were reproducible and easy to analyse (**Fig 2 and Table 3**). Our data suggest that 50 μg protein concentration gives an adequate quantity to have visually distinct spots and is suitable for analysing 2D-PAGE gel in the case of *Cajanus* species.

**Table 3:** Number of protein spots observed on 2D-PAGE gel.

Pigeon pea genotypes	No. of spots in 2D gel of total protein		No. of spots in 2D gel of chloroplast protein	
	250 μg loaded	50 μg loaded	250 μg loaded	50 μg loaded
ICPL-332	678 ± 08	220 ± 25	566 ± 10	132 ± 10
ICP-15738	597 ± 22	182 ± 18	486 ± 14	148 ± 16

### Optimal pH gradient for two-dimensional SDS-PAGE

The IPG strips are used as they contain stable pH gradients which enables the focusing of both acidic and basic proteins on a single strip. The narrow pH range gradient of IPG strips which was spread over a greater physical distance allows fine separation of proteins [39]. This spread allowed proteins with even narrow differences in isoelectric point (pI) values to get separated with higher resolution. To optimize the suitable pH gradient range for total and chloroplast proteins in *Cajanus* species we first resolved the protein on IPG strips (pH 3–10; 7 cm length; Bio-Rad, USA). We observed that in the case of total proteins, most of the proteins got separated within the pH range of 4.5–8. To get further separation, we used IPG strips (pH 4–7; 7 cm length; Bio-Rad, USA) and as expected the proteins appeared to be closely aligned in the IPG strip (pH 3–10). Proteins were adequately separated in the IPG strips with a pH range of 4 to 7 and individual spots were visible after staining (**Fig 2**).

### Improved silver staining process for visualization of protein bands

In the context of 2D-PAGE, the visualization of protein within the gel is predominantly achieved through the utilization of protein stains. The optimal selection of a stain necessitates a thoughtful evaluation of its inherent characteristics, constraints related to detection sensitivity, its efficacy in staining protein, considerations for subsequent applications, and compatibility with the available imaging equipment [40, 41]. Among various staining procedures available, the silver nitrate stain is one of the important stains used for the visualization of protein. Even though the silver staining protocol is to a certain extent time-consuming and complex, it can be about 100 times more sensitive than usual Coomassie Blue R-250 dye staining and able to detect highly glycosylated proteins in 2D-PAGE gels [42, 43]. We performed here two different methods of silver staining which we named as short/long protocol depending on the duration of protein fixation. We observed that in long long-duration method due to overnight fixation of proteins although a large number of protein spots are visible on the SDS-PAGE gel however due to overcrowding it’s become difficult to analyse and subsequently excise the protein spots from the gel [44]. In the case of short-duration protocol, although protein spot numbers were less, each spot was visible which enhanced its suitability for comparative analysis of differentially expressed proteins followed by gel excision. We found that in the case of Cajanus species total protein separation in a 2-D gel can be achieved by using a 7 cm IPG strip, loading 50 μg proteins, and performing the short duration of silver staining mentioned here are the best parameters (**Fig 2**). Thus, we have optimized a protocol for protein extraction suitable for both cultivated and wild genotypes of pigeon pea (**Table 4**). Additionally, for the first time, a highly efficient *Cajanus* species chloroplast protein extraction protocol suitable for proteomic research was also made available.

**Table 4:** Modification recommended for quality protein extraction and Isoelectric focussing using a 2-D PAGE method.

Critical steps	Published protocol (Singh *et al*., 2015)	Recommended protocol based on our findings
**Protocol I**	Extraction in Phosphate buffer followed by TCA-Acetone precipitation	Tris-Cl extraction followed by TCA-Acetone precipitation
**Extraction buffer composition**	1) 0.1 M Phosphate buffer, pH 7.5	1) 120 mM Tris-Cl, pH 8.0
	2) 0.07% β-ME	2) 200 mM NaCl
	3) Acetone (80%, w/v)	3) 2mM EDTA
	4) TCA (10%, w/v)	4) 0.07% β-ME
		5) 25 mM DTT
		6) Protease inhibitor cocktail (Sigma)
		7) Acetone (80%, w/v)
		8) TCA (10%, w/v)
**Protocol II**	Phenol extraction Method	Phenol extraction Method
**Extraction buffer composition**	1) 0.7 M Sucrose	In the modified extraction buffer 25 mM DTT in place of 0.07% β-ME and Protease inhibitor cocktail (Sigma) in place of 1 mM PMSF was recommended.
	2) 0.5 M Tris-Cl, pH 8.0	
	3) 30 mM HCl	
	4) 50 mM EDTA	
	5) 0.1 M KCl	
	6) 0.07% β-ME	
	7) 1 mM PMSF	
	8) 0.1 M Ammonium acetate in cold methanol	
**Washing step**	3 times	6 times
**IEF condition**	100 V for 1 h in step and hold, 500 V for 2 h in step and hold, 1000 V for 1 h in gradient mode, 8000 V for 2.30 h in gradient mode and 8000 V for 30 min. in step and hold	As mentioned in Table 1
**Equilibration buffer composition**	6 M urea, 75 mM Tris HCl, pH 8.8, glycerol 29.3%, SDS 2%, BPB 0.002% with 10 mg ml^−1^ DTT (Buffer I) & 25 mg ml^−1^ IAA (Buffer II)	6M Urea, 2%SDS, 0.375M Tris-Cl, 20% glycerol with 130mM DTT (Buffer I) & 135mM Iodoacetamide (Buffer II)
**Staining procedure**	Coomassie brilliant blue	Short protocol of silver staining

### Identification of proteins by MS analysis

The suitability of extracted protein for MS analysis was confirmed by the identification of protein spots. Ten differentially expressed protein spots were encircled and numbered (**Fig 2G**), selected from the gel of total protein of pH range 4–7, and analysed by MS/MS. The proteins were first digested with trypsin and then, after desalting, analysed with Orbitrap LC-MS/MS. The identification of proteins was done with Proteome Discoverer (v2.5) against the Uniprot *Cajanus cajan* database. All spots were successfully identified, characterized, and summarized (**Table 5**). The peptide identification had less than 1% FDR which means they were identified with high confidence. The coverage varied between 2 to 22%. The low coverage in some cases might be because of few peptides cannot fly well due to their size.

**Table 5:** List of identified proteins from pigeon pea total protein after MS analysis.

Spot No.	Accession no. (Uniprot)	Proteome Discoverer (v2.5) search result against the UniProt *Cajanus cajan* database	Function	PSM score	Protein coverage (%)	Peptide match	MW (kDa)	Calculated pI	Match with the organism
1	A0A151U2U8	3-isopropyl malate dehydratase	Leucine biosynthesis	1	3	1	50.4	5.9	*Cajanus cajan*
2	*A0A151T9N7*	NAC domain-containing protein 29	Plant-specific transcription factors	1	11	1	38.2	9	*Cajanus cajan*
3	*A0A151TZI8*	Retrotransposable element Tf2	Nucleic acid binding activity	1	3	1	106.1	8.88	*Cajanus cajan*
4	*A0A151SM41*	Retrovirus-related Pol polyprotein from transposon opus	Nucleic acid binding activity	1	6	1	75.7	9.01	*Cajanus cajan*
5	*A0A151S8N3*	Myosin-Vb	Cytoskeletal activity	1	4	1	107.6	7.94	*Cajanus cajan*
6	*A0A151T5W4*	DUF4219 domain-containing protein	Unknown	1	22	1	16.3	9	*Cajanus cajan*
7	*A0A151QMU8*	Retrovirus-related Pol polyprotein from transposon TNT 1–94	Nucleic acid binding activity	1	4	1	115.1	8.59	*Cajanus cajan*
8	A0A151RXC8	Retrovirus-related Pol polyprotein from transposon 17.6	Nucleic acid binding activity	1	2	1	38.4	9.47	*Cajanus cajan*
9	*A0A151TJK2*	Uncharacterized protein	Unknown	1	6	1	28.1	7.17	*Cajanus cajan*
10	*A0A151TSB7*	Alcohol dehydrogenase 1	Stress response, Seedling development	1	3	1	43.1	6.58	*Cajanus cajan*

### Recovery of intact pigeon pea chloroplast

**Fig 3:**
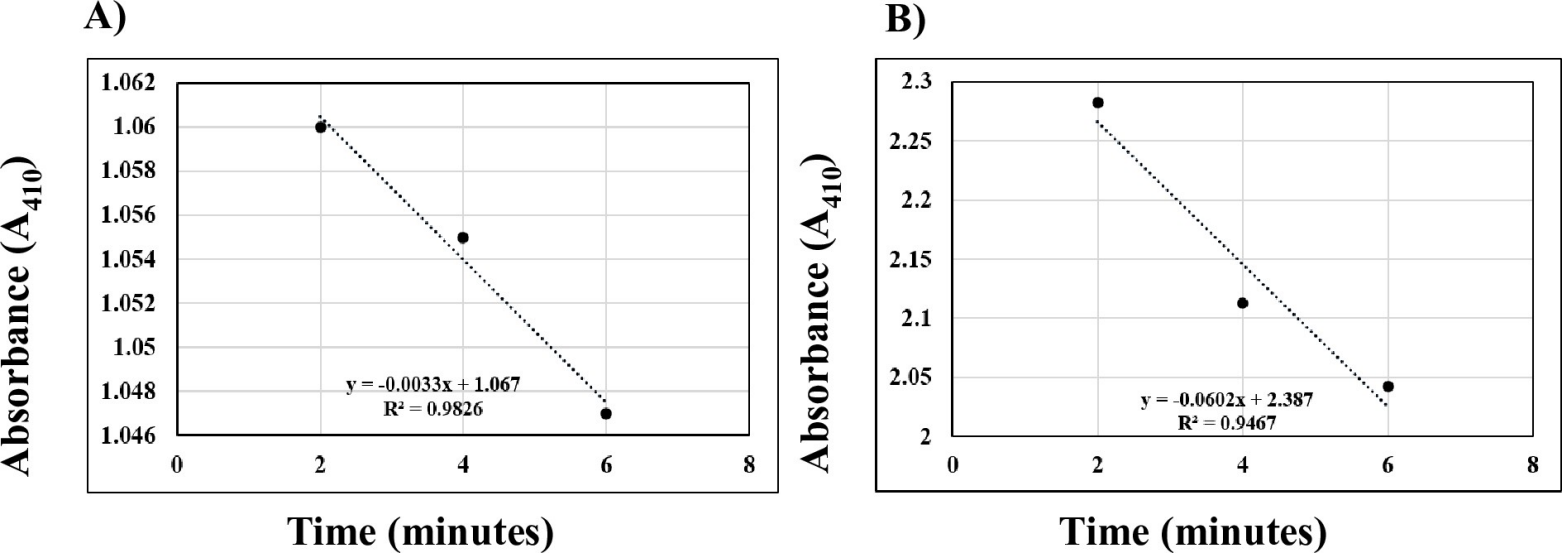
Ferricyanide photo reduction assay to analyse the quality of isolated intact chloroplast. A) Rate of decrease in absorbance (A_410_) per unit time measured without osmotic shock. B) Rate of decrease in absorbance (A_410_) per unit time measured with osmotic shock.

The most crucial step in performing proteomic analysis is to obtain high-quality proteins suitable for performing 2D-PAGE. Even though there are many cytosolic protein extraction protocols, reported in the past, from a diverse range of plant species but the same remained challenging in the case of chloroplast proteins extraction [19, 26, 45–50]. This is the first time we have optimized a chloroplast protein extraction protocol from *Cajanus* species suitable for proteomics research. The protocol described here provides an additional advantage as it overcomes the limitation of the conventional method which needs ultracentrifuge for the isolation of high-quality intact chloroplast. Using our established method similar results can be obtained even at low speeds of 3000–12000 rpm. All the steps for chloroplast isolation were performed in the dark to prevent the build-up of starch molecules which otherwise could rupture the membrane thereby reducing the yield of intact chloroplast [51]. The intact chloroplast was isolated from the interphase of two percoll gradients. The intactness of chloroplast was estimated from a ferricyanide photo reduction assay and a graph plotted with absorbance on the Y-axis against time on the X-axis (**Fig 3**). Around 94.52% of intact chloroplasts were recovered with our established protocol. The yield of the intact chloroplast was calculated on a unit chlorophyll basis (mg of chlorophyll) and around 13.56 mg of chlorophyll from 2 ml of chloroplast solution was obtained from 10g of leaf sample.

### Phenol-based extraction buffer for extraction of chloroplast protein

**Fig 4:**
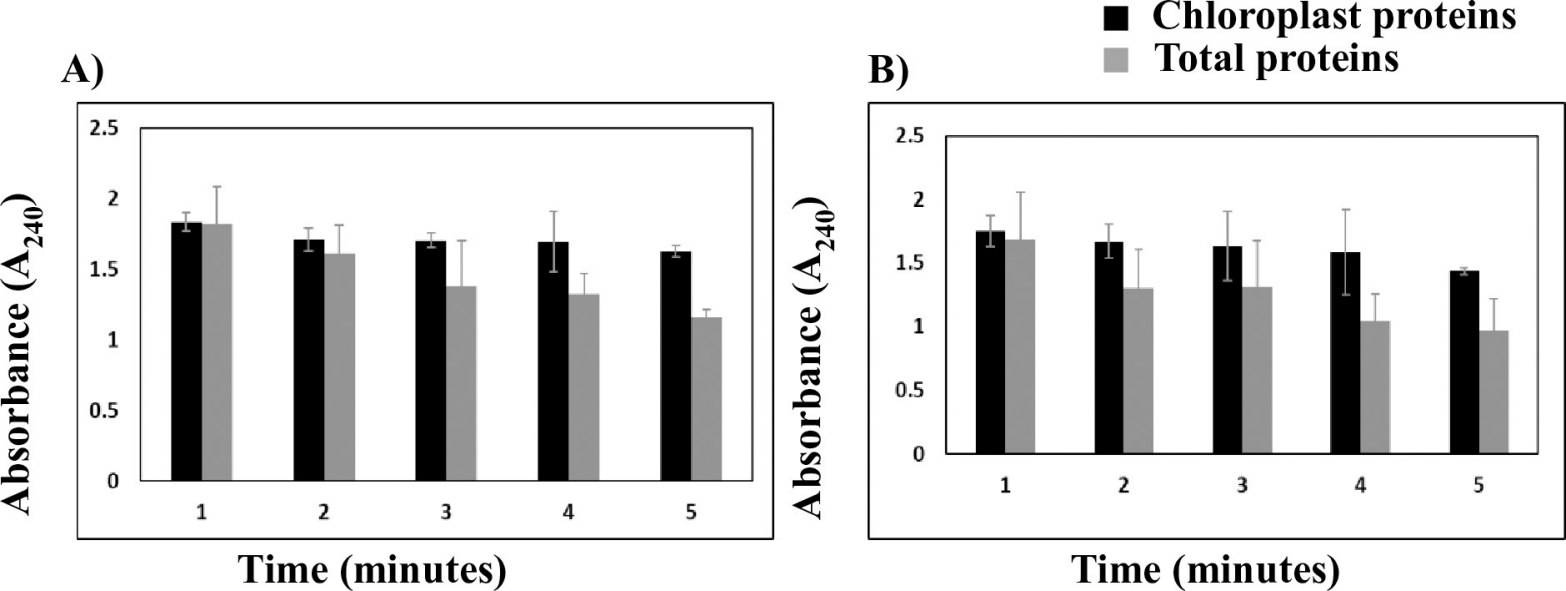
Catalase assay for determining the purity of chloroplast protein. A) Rate of decrease in absorbance (A_240_) per unit time was prominent in the fraction comprised of total proteins due to the presence of in vivo high catalase activity in comparison to the fraction comprised of chloroplast proteins isolated from wild type genotype *Cajanus cajan*. B) Rate of decrease in absorbance (A_240_) per unit time was prominent in the fraction comprised of total proteins due to the presence of *in vivo* high catalase activity in comparison to the fraction comprised of chloroplast proteins isolated from wild type genotype *Cajanus scarabaeoides*.

From 10 g of fresh leaf sample around 1.33 ± 0.25 mg/gm and 0.88 ± 0.19 mg/gm chloroplast protein was isolated in the case of *C*. *cajan* and *C*. *scarabaeoides* respectively. The purity of the extracted chloroplast protein was first determined through catalase enzyme assay. We found that in cytosolic protein fraction usually, the catalase enzyme activity was higher which is evident from the gradual reduction in the absorbance (A_240_) with time in the presence of H_2_O_2_ as a substrate (**Fig 4**). However, the chloroplast fraction didn’t show any significant reduction in absorbance due to the absence of cytosolic protein contaminants. Hence, the catalase assay confirmed that the extracted chloroplast proteins contain negligible levels of contamination from the cytosolic protein. It was observed that the total protein from *C*. *scarabaeoides* turned brownish without the addition of any reducing agents in the protein extraction buffer due to high phenolic content. These phenolic compounds in young tissues are mainly present in vacuoles [52] and can be removed by the addition of strong reducing agents like DTT and PIC in the extraction buffer. In contrast, chloroplast protein was observed as a white color pellet without the addition of any reducing agent. This could be because we extracted chloroplast protein directly after the isolation of chloroplast which does not contain phenolic compounds. Further, the isolated total and chloroplast proteins were electrophoresed over 12% SDS-PAGE gel to observe the difference in the pattern of bands. We observed that in the lane corresponding to chloroplast proteins significant number of bands were missing when compared with the lane corresponding to total proteins. These results indicated that we were able to remove the majority of cytosolic proteins from the chloroplast protein fraction. Thus, our data suggest that the extracted chloroplast proteins are of high quality and are suitable for performing proteomic analysis.

### Presence of light-harvesting complex protein in the chloroplast protein fraction

**Fig 5:**
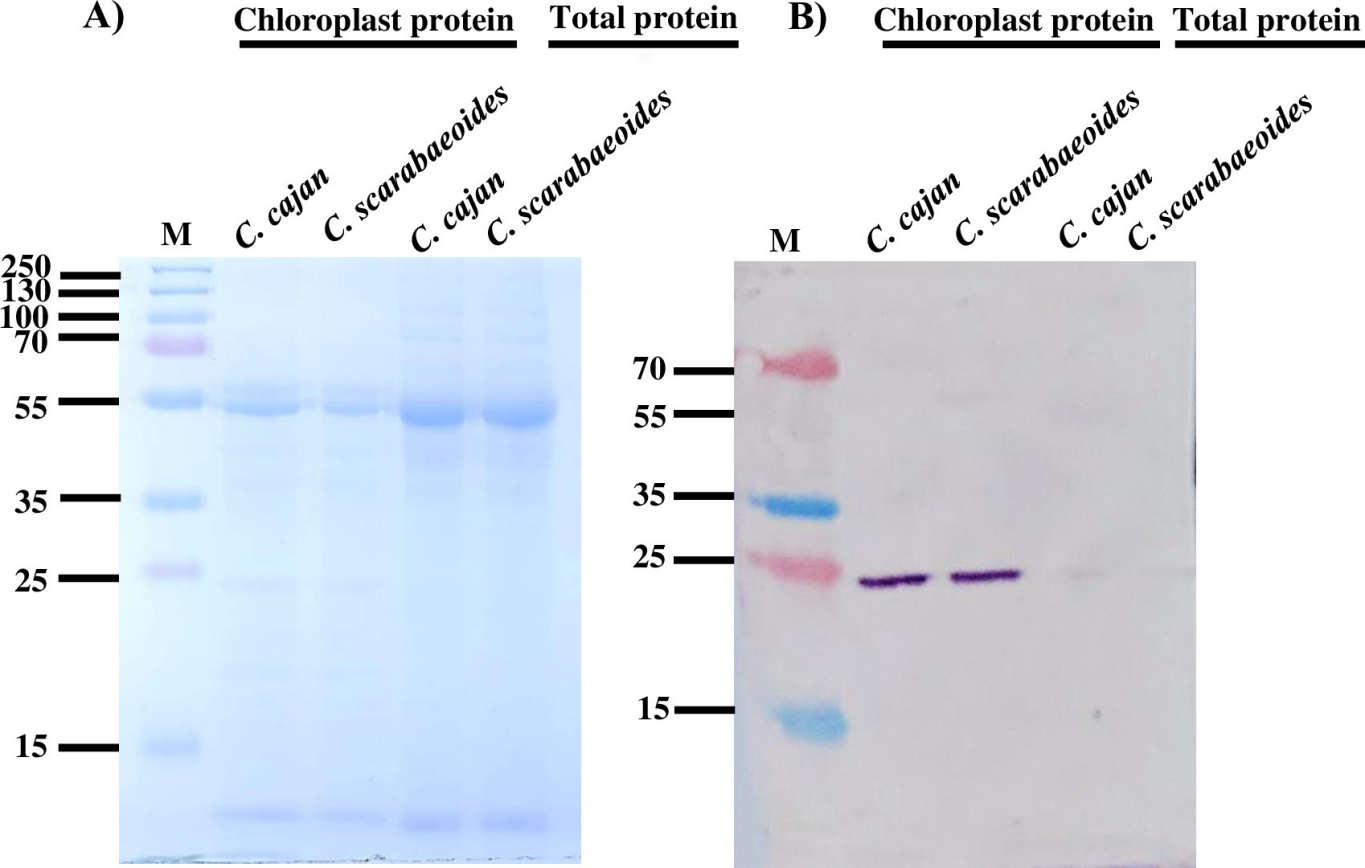
SDS-PAGE and immunoblot analysis of the proteins to determine the purity of extracted chloroplast protein. (A) One dimensional SDS-PAGE analysis of total and chloroplast proteins from pigeon pea genotype *C*. *cajan* and *C*. *scarabaeoides* showing absence of significant number of cytosolic proteins in chloroplast protein fraction in comparison to total protein fraction. (B) Immunoblot analysis of chloroplast and total protein, using LHCb1/LHCⅡ type I chlorophyll a/b binding protein antibody, showing higher band intensity in the proteins extracted from chloroplast fraction in comparison to the one from total proteins. Equal loading of the protein was confirmed with ponceau staining and anti- LHCb1/LHCⅡ type I was used at a concentration of 1:2500.

To determine the enrichment of the chloroplast proteins from the isolated chloroplast in comparison to the total protein fraction, we performed immunoblot analysis with chloroplast specific LHCb1/LHCⅡ type Ⅰ chlorophyll a/b binding protein antibody. We observed the presence of a higher amount of chlorophyll a/b binding proteins in the chloroplast protein fraction from *C*. *scarabaeoides* and *C*. *cajan* in comparison to the total protein fraction (**Fig 5**). Immunoblot analysis with the antibody against the light-harvesting complex suggests enrichment of the chloroplast proteins in the fraction comprising the intact chloroplast. Altogether, the immunoblot analysis indicates that the isolated chloroplast is of high quality with a negligible amount of cytosolic protein contamination and hence, can be used for any downstream proteomic analysis.

### Two-dimensional SDS-PAGE analysis of chloroplast proteins

**Fig 6:**
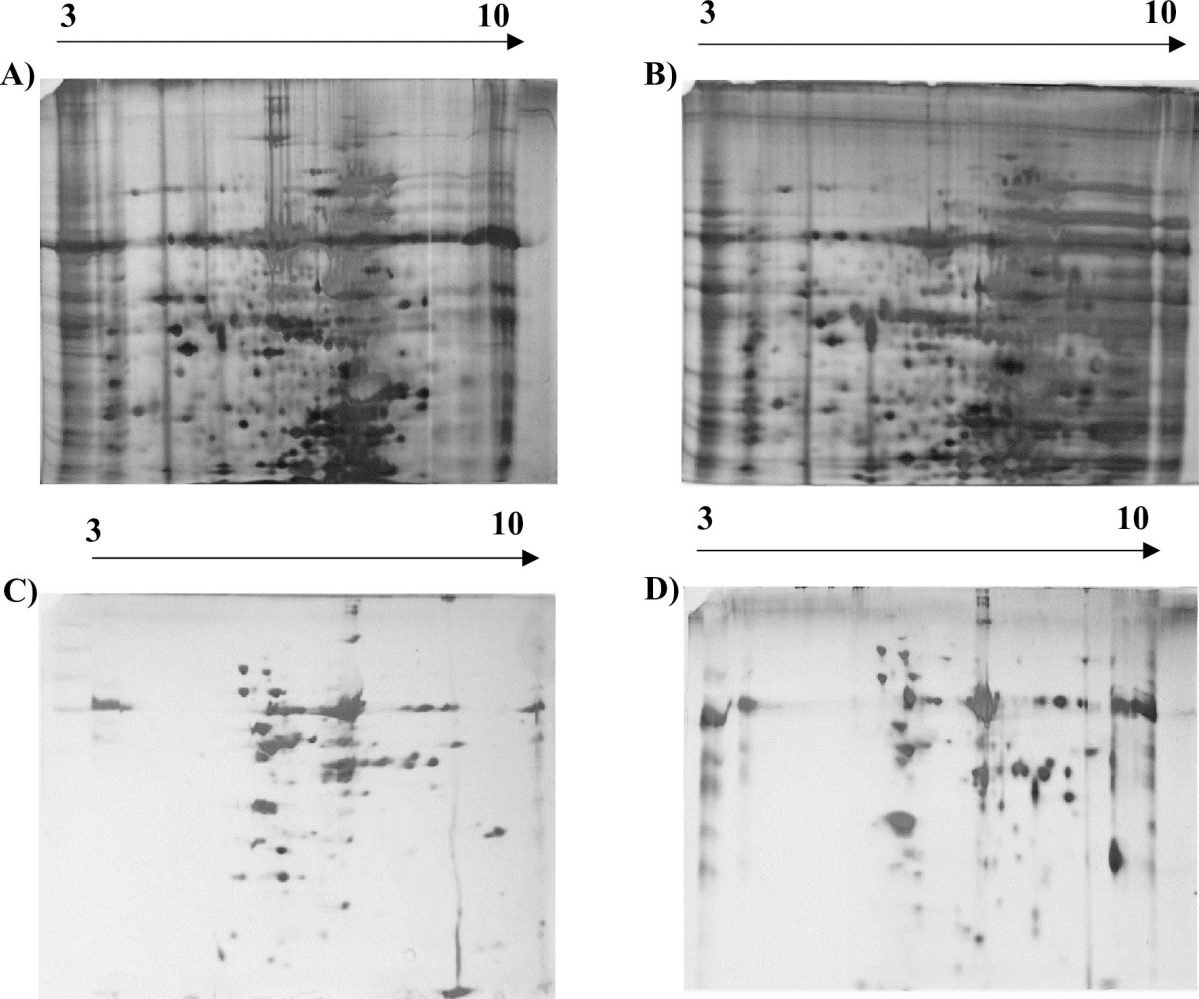
Two dimensional SDS-PAGE analysis of chloroplast proteins from pigeon pea genotypes *C*. *scarabaeoides* and *C*. *cajan*. A-B) Around 250 μg of chloroplast proteins extracted from pigeon pea genotypes *C*. *cajan* and *C*. *scarabaeoides* were resolved during IEF over IPG strip (pH gradient range of 3–10) and then electrophoresed on 12% SDS-PAGE gel. C-D) Chloroplast protein load was reduced to 50 μg in both pigeon pea genotypes of *C*. *cajan* and *C*. *scarabaeoides* due to the lack of visually distinct spots at high concentration and were resolved during IEF over IPG strip (pH gradient range of 3–10) and electrophoresed on 12% SDS-PAGE gel, for staining of all gels short protocol of silver staining was used.

From the extracted chloroplast proteins, we initially resolved 250 μg proteins over IPG strips but due to the appearance of distinct total chloroplast protein spots in the concentration to 50 μg. We observed that the proteins of the chloroplast fraction accumulated in a pH range of 6–10 when resolved over the IPG strips (pH 3–10; 7 cm length; Bio-Rad, USA). Our results showed that the pH range of 3–10 rather than the pH range of 4–7, is suitable for separation and adequate visualization of chloroplast protein on 2D-PAGE after staining (**Fig 6**). Our protocol is highly efficient for the isolation of intact chloroplast followed by the extraction of high-quality chloroplast protein suitable for performing any proteomic research.

## Conclusion

In the current study, we established a method for the extraction of total proteins and another method for chloroplast protein extraction from cultivated and wild genotypes of pigeon pea. The existing method yielded a very low amount of protein in these genotype*s* due to the elevated level of tannins and phenolic compounds [17]. The extracted proteins showed poor separation during IEF accompanied by multiple horizontal/vertical streaks making it unsuitable for performing any proteomic analysis. To overcome these issues, we used 25 mM DTT along with a protease inhibitor cocktail in the extraction buffer and recovered 2-fold more protein yield in comparison to the original method. In our case, by increasing the number of washing steps of protein pellets from 3 to 6 with acetone reduced the contamination with chlorophyll and salts present in the precipitation buffer. Further, we observed proper separation of proteins during IEF and 2D-PAGE gels without any horizontal/vertical streaks. Although the modified method does not show any difference in the net yield of total proteins extracted in comparison to the original method in cultivated genotype *C*. *cajan*, the modification resulted in improved protein separation during IEF and reduction of horizontal/vertical streaks.

We, also optimized for the first time a highly efficient and reproducible protocol for the isolation of intact chloroplast without ultracentrifugation step followed by protein extraction for performing 2D-PAGE in pigeon pea genotype *C*. *cajan* and *C*. *scarabaeoides*. Ferricyanide photo reduction assay and catalase assay confirmed the intactness of the isolated chloroplast and purity of extracted proteins respectively. Immunoblotting and electrophoresis gave further confirmation about the enrichment of chloroplast proteins with a negligible amount of contamination from the cytosolic proteins. Our data suggest that we were able to optimize an efficient, robust, and reproducible protocol for the isolation of high-quality intact chloroplast followed by extraction of proteins suitable for performing proteomic analysis.

## Supporting information

S1 Fig Vegetative growth stage of pigeon pea genotypes *C*. *scarabaeoides* and *C*. *cajan* photographed at different time points. (DOCX)

S2 Fig Two dimensional SDS-PAGE analysis of total and chloroplast proteins from *C*. *scarabaeoides* before optimization of the composition of protein extraction buffer. (DOCX)

S1 Data (XLSX)
