# Supplementary material for: Improved methods for total and chloroplast protein extraction from Cajanus species for two-dimensional gel electrophoresis and mass spectrometry
Source: PLoS One. 2024 Aug 15;19(8):e0308909. doi: 10.1371/journal.pone.0308909 (PMC11326652; doi:10.1371/journal.pone.0308909)
Supplement: S1 Fig — (DOCX) [file pone.0308909.s001.docx]

**Supporting information**

**S1 Fig. Vegetative growth stage of pigeon pea genotypes *C. scarabaeoides* and *C. cajan* photographed at different time points**

A-B) Lateral view of *C. scarabaeoides* (ICP-15738) and *C. cajan* (ICPL-332) of pigeon pea at 35 days after germination (DAG)

C-D) Lateral view of *C. scarabaeoides* (ICP-15738) and *C. cajan* (ICPL-332) of pigeon pea at 70 days after germination (DAG)

E-F) Top view of *C. scarabaeoides* (ICP-15738) and *C. cajan* (ICPL-332) of pigeon pea at 35 DAG

**
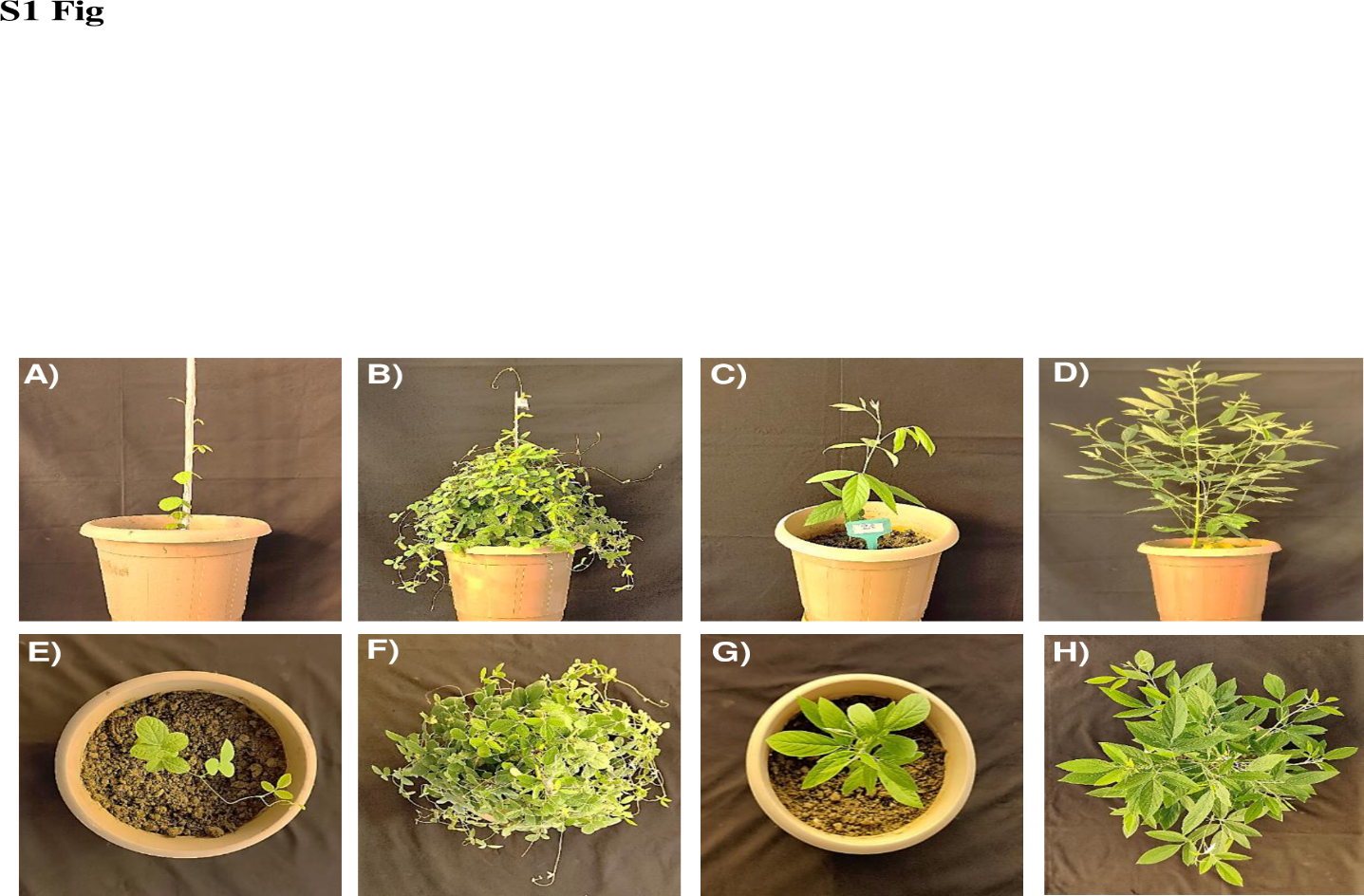
**G-H) Top view of *C. scarabaeoides* (ICP-15738) and *C. cajan* (ICPL-332) of pigeon pea at 70 DAG
