# Supplementary material for: Improved methods for total and chloroplast protein extraction from Cajanus species for two-dimensional gel electrophoresis and mass spectrometry
Source: PLoS One. 2024 Aug 15;19(8):e0308909. doi: 10.1371/journal.pone.0308909 (PMC11326652; doi:10.1371/journal.pone.0308909)

**S2 Fig. Two dimensional SDS-PAGE analysis of total and chloroplast proteins from *C. scarabaeoides* before optimization of the composition of protein extraction buffer**

1. Around 100 µg of total proteins extracted from pigeon pea genotype *C. scarabaeoides* were resolved during IEF over IPG strip (pH gradient range of 3-10) and then electrophoresed on 12% SDS-PAGE gel
2. Around 100 µg of chloroplast proteins extracted from pigeon pea genotype *C. scarabaeoides* were resolved during IEF over IPG strip (pH gradient range of 3-10) and then electrophoresed on 12% SDS-PAGE gel


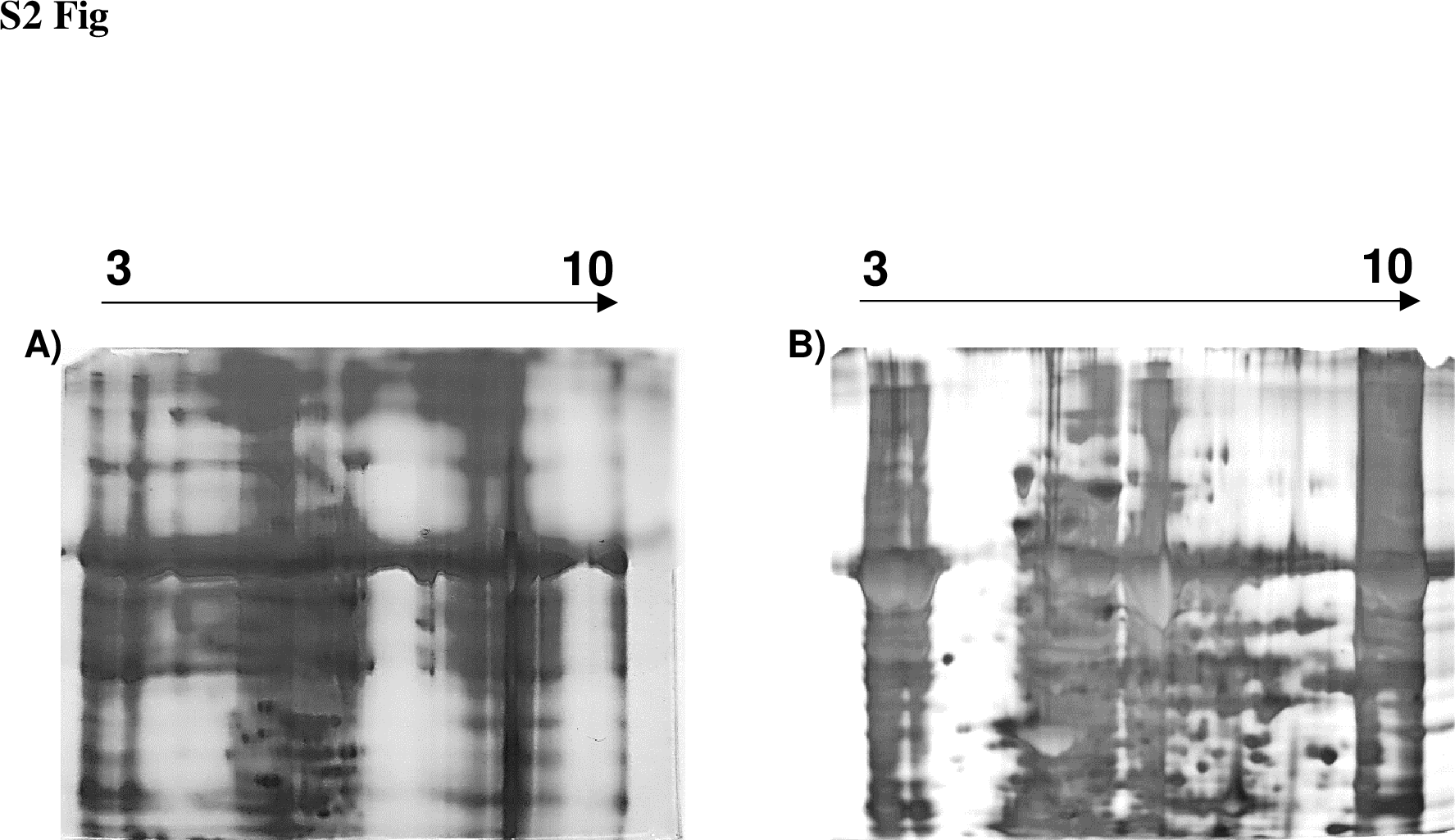

Supplement: S2 Fig — (DOCX) [file pone.0308909.s002.docx]
